# Quantitative assessment by measurement and modeling of mobile target elongation in cone‐beam computed tomographic imaging

**DOI:** 10.1120/jacmp.v15i3.4634

**Published:** 2014-05-08

**Authors:** Imad Ali, Nesreen Alsbou, Ozer Algan, Terence Herman, Salahuddin Ahmad

**Affiliations:** ^1^ Department of Radiation Oncology University of Oklahoma Health Sciences Center Oklahoma City OK USA; ^2^ Department of Electrical and Computer Engineering Ohio Northern University Ada OH USA

**Keywords:** cone‐beam CT, respiratory motion, image artifacts, motion model, mobile target, elongation

## Abstract

The purpose of this study was to assess quantitatively elongation of mobile targets in cone‐beam CT (CBCT) imaging by measurement and modeling. A mathematical model was derived that predicts the measured lengths of mobile targets and its dependence on target size and motion patterns in CBCT imaging. Three tissue‐equivalent targets of differing sizes were inserted in an artificial thorax phantom to simulate lung lesions. Respiratory motion was mimicked with a mobile phantom that moves in one‐dimension along the superior‐inferior direction at a respiration frequency of 0.24 Hz for eight different amplitudes in the range 0‐40 mm. A mathematical model was derived to quantify the variations in target lengths and its dependence on phantom motion parameters in CBCT. Predictions of the model were verified by measurement of the lengths of mobile targets in CBCT images. The model predicts that target lengths increased linearly with increase in speed and amplitude of phantom motion in CBCT. The measured lengths of mobile targets imaged with CBCT agreed with the calculated lengths within half‐slice thickness spatial resolution. The maximal length of a mobile target was independent of the frequency and phase of motion. Elongation of mobile targets was similar in halffan and full‐fan CBCT for similar motion patterns, as long as the targets remained within the imaging view. Mobile targets elongated linearly with phantom speed and motion amplitude in CBCT imaging. The model introduced in this work assessed quantitatively the variation in target lengths induced by motion, which may be a useful tool to consider elongations of mobile targets in CBCT applications in diagnostic imaging and radiotherapy.

PACS number: 87.57.qp

## I. INTRODUCTION

CBCT imaging applications have increased significantly over the last decade. New imaging systems with high resolution two‐dimensional, flat‐panel detectors^(1)^ were introduced that provide robust tools to acquire volumetric CT imaging. In radiotherapy, on‐board kV imaging systems that can perform CBCT have been integrated with dose delivery machines that are employed to perform image‐guided radiation therapy.^(2)^ CBCT imaging is finding potential application in diagnostic imaging^(3)^ and image‐guidance of dental^(4)^ and surgical^(5)^ procedures. Patient motion causes substantial image artifact in CBCT images.^(6)^ In CBCT, larger volumes with many slices are acquired simultaneously with larger imaging views of the projections, in comparison with fan‐beam CT where slices are acquired sequentially in snapshots.^(7)^

Various approaches were employed to reduce motion artifacts in CBCT imaging that include motion correction in projections prior to CBCT reconstruction^8^, ^9^ where the pixels of mobile phantom are mapped to a stationary position by applying shifts reverse to the direction of motion. One limitation of this technique is that all pixels in a projection are shifted equally, assuming that all phantom voxels move similarly which is not accurate in real patient motion. Four‐dimensional CBCT is used to overcome motion artifacts, in which the projections are sorted in various respiratory phases^10^, ^11^ and used to reconstruct CBCT images specific for each respiratory phase.^(12)^ Although, motion artifacts are reduced in 4D CBCT, fewer number of projections sorted in the different respiratory phases are employed in image reconstructions, which increase noise and may lead to severe streaking artifacts. In this work, lengths of mobile targets in CBCT imaging were assessed quantitatively by measurement and modeling. A mathematical model was introduced that calculates the apparent lengths of mobile targets in CBCT imaging. This model predicts the dependence of elongation on initial static length and motion parameters such as amplitude and speed of a mobile target. The predictions of the model were verified with measurements of the lengths of three targets embedded in a phantom that simulate lung lesions and moved in one‐dimension along superior‐inferior direction.

## II. MATERIALS AND METHODS

### A. Phantom setup and imaging

**Figure 1:**
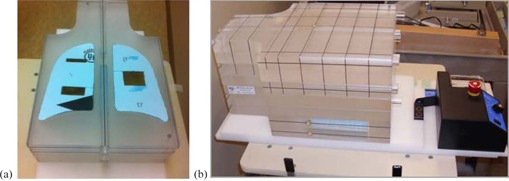
Three mobile targets (a): small $\left(3\times 5\times 1\;{\text{cm}}^{3}\right)$ at superior right side, medium $\left(3\times 5\times 2\;{\text{cm}}^{3}\right)$ at inferior right side, and large $\left(3\times 5\times 4\;{\text{cm}}^{3}\right)$ at medial right side inserted in a low‐density foam. Mobile platform (b) with phantom.

A mobile phantom was assembled from three targets with different sizes: a small $\left(3\times 2\times 5\;{\text{cm}}^{3}\right)$, medium $\left(3\times 1\times 5\;{\text{cm}}^{3}\right)$, and large $\left(3\times 4\times 5\;{\text{cm}}^{3}\right)$ fabricated from water‐equivalent gels. The targets were then embedded in low‐density foam slab equivalent to lung tissue, as shown in Fig. 1(a). The foam slab was inserted between Solid Water phantom slabs which were mounted on a moving platform (Standard Imaging, Inc., Middleton, WI), as shown in Fig. 1(b). The phantom was imaged using an on‐board imager (OBI) with kV CBCT that was installed on a TrueBeam linear accelerator (Varian Medical Systems, Inc., Palo Alto, CA). During CBCT imaging, the phantom was moved cyclically in the superior‐inferior direction with different motion amplitudes: 0, 5, 10, 15, 20, 25, 30, and 40 mm and a frequency of 15 cycles per minute. CBCT images were acquired using the half‐fan (125 kVp, 264 mAs) and full‐fan (100 kVp, 146 mAs) imaging modes with a slice thickness of 2 mm. The standard thorax technique was used for half‐fan CBCT scans where projections were acquired approximately over a full circle for one minute. The head standard technique was used for full‐fan CBCT scans in which the projections were collected over half a circle in nearly half a minute. The CBCT images were processed with the Eclipse treatment planning system (Varian Medical Systems). The distance measurement tools in Eclipse were used to measure the lengths of the three targets on in‐plane coronal views from all acquired scans when the phantom was stationary and mobile. The CT number level and window were adjusted to enhance the image contrast of the mobile targets relative to the lung tissue‐equivalent to outline the boundaries of blurred targets in CBCT images.

### B. Modeling of target elongation in CBCT

**Figure 2:**
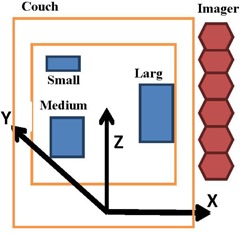
Schematic of the reference coordinates of the imaging system.

The couch is stationary during CBCT imaging from the kV OBI. The imaging frame is given by reference coordinates (X, Y, Z), as shown in Fig. 2. If the phantom is moving during imaging, $V_{P}$, then the speed of a mobile target in the CBCT imaging frame is given by the following equation:

(1) $$V_{z}^{\text{Im}g}(t)=V_{P}(t)$$

The target length in the imaging frame will be elongated and its apparent length in the imaging frame is given by the following equation:

(2) $$L=L_{o}+V_{P}T$$

where *T* is the time interval that the target remains within the imaging view.

If a target moves cyclically with an amplitude, *A*, and frequency, *f*, along the superior‐inferior direction (z axis) as shown in Fig. 2, then the position of a voxel at $Z_{o}$ in a mobile target varies with time, t, as given by the following equation:

(3) $$Z(t)=Z_{o}+A\text{sin}(\omega t+\varphi )$$

where ω=2πf and φ represents the phase angle at t=0. The speed of point Z in the imaging frame is obtained by the first derivative of Eq. (4), as given by the following:

(4) $$V_{P}(t)=\frac{dZ(t)}{dt}=\omega A\text{cos}(\omega t+\varphi )$$

In the case of varying speeds from sinusoidal respiratory motion and by substituting Eq. (4) in Eq. (2), the time varying length of the target is proportional to amplitude and frequency of motion as given by the following:

(5) $$L(t)=L_{o}+[\omega A\text{cos}(\omega t+\varphi )]t$$

where *t* is time with the target in the imaging view.

In CBCT imaging, the scanning time required to perform a CBCT scan ranges from 30 to 60 sec, which is longer than the period (4 sec) of the cyclic motion. Therefore, the voxels within the target will extend back and forth several times from bottom to peak, (−A,A) along the motion track (z axis) during CBCT scanning. By integration of the length in Eq. (5), the target length will be extended and its maximal length is given by the following equation:

(6) $$L=L_{o}+2A$$

In order to test the previous modeling of the target elongation in CBCT images, MATLAB (MathWorks, Inc., Natick, MA) coding was used to calculate the length of the three mobile targets. The measured lengths were then compared with the calculated lengths from modeling. The target elongation in CBCT imaging was calculated considering one‐dimensional constant speeds and cyclic motions along the superior‐inferior direction according to Eqs. (2) and (6), respectively.

## III. RESULTS

**Figure 3:**
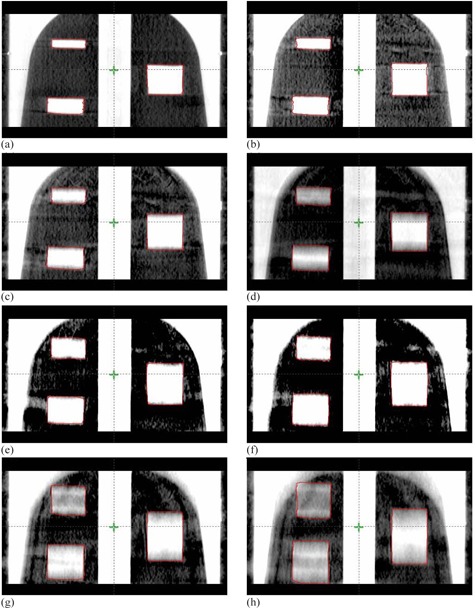
Coronal views of the phantom with the three inserted targets (small, medium, and large) and different motion amplitudes using half‐fan CBCT images. The phantom moved along the superior‐inferior direction (z axis) sinusoidally with eight different ranges of motion (ROM is equal two motion amplitudes): 0, 5, 10, 15, 20, 25, 30, and 40 mm with a single respiration frequency of 15 cycles per minute (a)‐(h), respectively.

**Figure 4:**
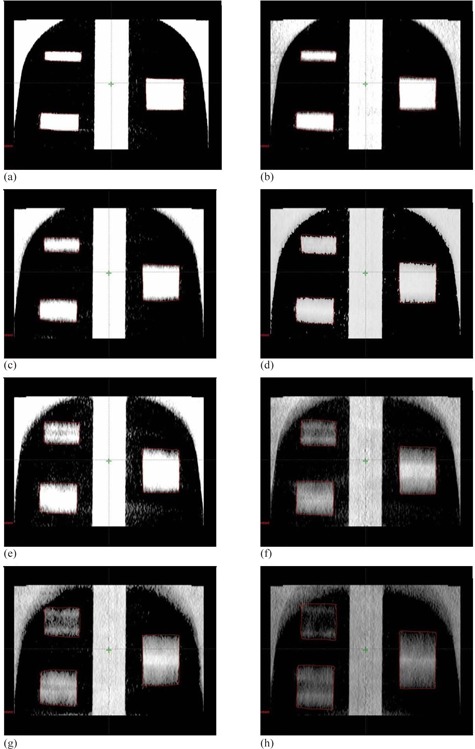
Coronal views of the phantom with the three mobile targets (small, medium, and large) moving with motion patterns as in Fig. 2 using full‐fan CBCT.

Figure 3 shows coronal views of the phantom with the three targets (small, medium, and large) for different motion amplitudes using half‐fan CBCT imaging. The lengths of the targets in the CBCT images increased linearly with the increase in the motion range, as shown in the coronal images in Figs. 3(a)‐(h). The motion phase was not controlled and the target could be at any position in the motion cycle during CBCT imaging and the images in Fig. 3 were acquired for one motion frequency of 15 cycles per minute. The maximal increase in the lengths of the various targets was independent of the frequency and phase of motion. The CT numbers were smeared over a longer spatial range differentially with the increase in ROM. Similar elongation motion artifacts were observed in full‐fan CBCT, as shown in Fig. 4.

**Figure 5:**
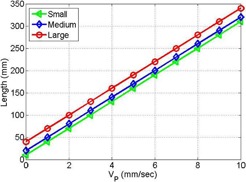
Length of mobile target as a function of the speed for the small, medium, and large targets considering 30 sec of imaging time.

Figure 5 shows lengths of the targets as a function of speed of the mobile phantom and time required for scanning according to Eq. (2). The length increases linearly with the speed of the phantom, as shown in Fig. 5. This is assumes that the target stays all the time (30 sec) within the cone‐beam imaging view.

**Figure 6:**
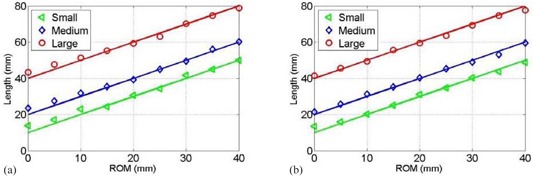
The data points represent the measured apparent lengths as a function of ROM for the small, medium, and large targets using half‐fan (a) and full‐fan (b) CBCT images. The solid lines represent the predictions of the motion model in CBCT imaging.

Figures 6 (a) and (b) represent the variations in lengths along z axis of the small, medium, and large targets as a function of ROM for half‐fan CBCT and full‐fan CBCT, respectively. The measured apparent lengths of the different targets increased linearly with the ROM of the respiratory phantom motion in agreement with predictions of Eq. (6) represented in the solid lines. The lengths of different targets measured from the CBCT images with the stationary phantom (ROM=0) where within 2 mm with the actual lengths of the small, medium, and large targets. This resulted mainly from the blurring of the targets at the superior and inferior ends of the targets due to the slice thickness of 2 mm which limits the spatial resolution of the length measurements. The accuracy of measuring the lengths of the mobile targets was limited by the slice thickness of acquired CBCT (±mm).

## IV. DISCUSSION

Measurements of the target lengths in CBCT show that the targets elongate linearly with the ROM of the mobile phantom in agreement with the prediction of the model. as given by Eq. (6).

This is due to the long image acquisition time (30 to 60 sec) compared to the period of cyclic phantom motion (4 sec) and large imaging view, where the full extent of the range of motion is imaged with CBCT from the kV on‐board imager. Image artifacts induced by phantom motion were similar in full‐fan and half‐fan CBCT modes because the targets were located to one side or the other of the phantom and the images of the targets were captured in all projections as the mobile phantom was scanned. However, if a target was in a central position in the imaging view where it will be imaged partially by the half‐fan scanning mode, then more complicated image artifacts may be induced by motion because the target will be missed in some projections during the scanning process.

The modeling of mobile target elongation for CBCT presented in this work is derived from basic physics principles for water‐equivalent homogenous targets moving in one direction considering a stationary imaging couch. The modeling addresses different motion patterns including mobile targets with constant speeds and cyclic motions imaged with an on‐board kV imaging system. However, this mathematical model does not apply for imaging systems with a moving couch, such as helical or axial CT.^7^, ^13^ Previous studies reported motion artifacts in CT imaging that include measurement and modeling of upper and lower bounds on mobile tumor elongation^(14)^ and empirical formulism of impact of motion artifacts on target volumes,^(15)^ and a quantitative theoretical model was developed that quantifies motion artifacts and relationships with imaging couch motion in helical CT.^(16)^ However, the theoretical modeling in this study assesses quantitatively the lengths of mobile targets specifically imaged with CBCT from kV on‐board imaging system.

The clinical implications of this study are similar to 4D CT imaging^10^, ^11^ where it is used to manage patient motion by establishing a relationship between the spread‐out of mobile tumors and patient motion parameters such as amplitude, phase, and frequency. However, several studies have reported that residual motion artifacts can still persist even in 4D CT imaging, which is due to misalignment or motion blurring artifact.^17^, ^18^ 4D CT can be impaired by irregular patient breathing.^(19)^ Mobile target broadening due to respiratory motion or other patient motions may cause serious misleading data in diagnostic CBCT images. In radiotherapy, erroneous representation of the gross tumor volumes, which is the visible area of a target, may cause inaccurate outlining of the primary tumor and nearby critical structures and normal tissue. The modeling developed in this work can be used to extract the range of elongation of a mobile target which can be used to define accurate margins for the treatment planning volume and perform accurate patient setup and tumor localization using image‐guidance with CBCT imaging. One limitation of this model is that it does not account for CBCT number variations induced by motion, which is under investigation in a future work.

## V. CONCLUSIONS

In this work, the elongation of mobile targets in CBCT imaging from kV on‐board imaging systems was quantified using both measurements and modeling. The mathematical model predicted quantitatively the variations in target lengths and their dependence on the motion parameters considering one‐dimensional motion in CBCT. The lengths of mobile targets elongated linearly proportional to the phantom speed or motion range. This modeling of the elongation of mobile targets may be employed to quantitatively assess the length and volume variations of mobile targets imaged with CBCT in diagnostic imaging and radiotherapy applications.
